# Intact neural representations of affective meaning of touch but lack of embodied resonance in autism: a multi-voxel pattern analysis study

**DOI:** 10.1186/s13229-019-0294-0

**Published:** 2019-11-27

**Authors:** Haemy Lee Masson, Ineke Pillet, Steffie Amelynck, Stien Van De Plas, Michelle Hendriks, Hans Op de Beeck, Bart Boets

**Affiliations:** 1 Department of Brain and Cognition, KU Leuven, Tiensestraat 102, box 3714, 3000 Leuven, Belgium; 2 Center for Developmental Psychiatry, KU Leuven, 3000 Leuven, Belgium; 3 Leuven Autism Research consortium, KU Leuven, 3000 Leuven, Belgium

**Keywords:** Autism spectrum disorder, Embodied simulation, Multi-voxel pattern analysis, Social touch aversion, Social touch observation, Theory of mind

## Abstract

**Background:**

Humans can easily grasp the affective meaning of touch when observing social interactions. Several neural systems support this ability, including the theory of mind (ToM) network and the somatosensory system linked to embodied resonance, but it is unclear how these systems are affected in autism spectrum disorder (ASD). Individuals with ASD exhibit impairments in the use of nonverbal communication such as social and reciprocal touch. Despite the importance of touch in social communication and the reported touch aversion in ASD, surprisingly little is known about the neural systems underlying impairments in touch communication in ASD.

**Methods:**

The present study applies a dynamic and socially meaningful stimulus set combined with functional magnetic resonance imaging (fMRI) to pinpoint atypicalities in the neural circuitry underlying socio-affective touch observation in adults with ASD. Twenty-one adults with ASD and 21 matched neurotypical adults evaluated the valence and arousal of 75 video fragments displaying touch interactions. Subsequently, they underwent fMRI while watching the same videos. Using multi-voxel pattern analysis (MVPA) and multiple regression analysis, we examined which brain regions represent the socio-affective meaning of observed touch. To further understand the brain-behavior relationship, we correlated the strength of affective representations in the somatosensory cortex with individuals’ attitude towards social touch in general and with a quantitative index of autism traits as measured by the Social Responsiveness Scale.

**Results:**

Results revealed that the affective meaning of touch was well represented in the temporoparietal junction, a core mentalizing area, in both groups. Conversely, only the neurotypical group represented affective touch in the somatosensory cortex, a region involved in self-experienced touch. Lastly, irrespective of the group, individuals with a more positive attitude towards receiving, witnessing, and providing social touch and with a higher score on social responsivity showed more differentiated representations of the affective meaning of touch in these somatosensory areas.

**Conclusions:**

Together, our findings imply that male adults with ASD show intact cognitive understanding (i.e., “knowing”) of observed socio-affective touch interactions, but lack of spontaneous embodied resonance (i.e., “feeling”).

## Background

Interpersonal touch, such as a hug or a slap, is a potent non-verbal communicative tool for expressing one’s emotions and intentions [1, 2]; thus, an appropriate understanding of the meaning of touch is crucial for social functioning. Humans can extract a vast amount of information, including other’s affective states, when merely watching a touch interaction [3, 4]. Identifying other’s emotions from these social cues involves a sophisticated neural circuitry, including the extended visual system, the limbic system [5], and regions implicated in social cognition [6].

Pertaining to social cognition, two complementary theoretical frameworks—along with their associated neural modules—have targeted the processing of emotional body language. The first aligns with the more cognitively oriented *theory of mind account* (ToM; both the modular-theory and theory-theory) and postulates that humans are able to infer other’s mental states (i.e., emotions, intentions, and beliefs) by means of meta-perspective reasoning [7–9]. The modular account postulates that ToM is achieved by an innate neural mechanism selectively involved in mental state inference [8]. The theory-theory account postulates that children are born with “naive” internal theories about the social world that are constantly revised in response to accumulated experiences, resulting in conceptual advances in mental state inference [10]. In both accounts, the ToM system has been depicted as a relatively effortful, controlled, and cognitively demanding form of social cognition [11], implicating the bilateral temporoparietal junction (TPJ) [12, 13]. The second theoretical framework originates from the *embodied simulation/resonance* literature, aligns with the mirror neuron mechanism theory, and posits that individuals implicitly infer other people’s emotional states from social cues by automatically re-enacting pre-acquired sensory experiences [14, 15].

While this second line of research initially focused on the observation of fairly simple motor activities, implicating the premotor cortex and inferior parietal areas [16, 17], more recent studies have started investigating the observation of simple touch [5, 18, 19] and more complex interpersonal touch [20]. Concerning touch observation, accumulating evidence suggests that activated brain regions go beyond the visual cortex and include somatosensory regions involved in the processing of self-experienced touch [18, 21–23]. This direct mapping of other’s bodily experiences to the self may aid in simulating and empathizing with others’ emotions (e.g., the pain we feel when we observe another person being injected with a needle). Accordingly, the level of activation in the somatosensory system during touch observation has been associated with interindividual differences in empathy [24–27].

Although many studies have affirmed the presence of interindividual differences in social cognition, the behavioral and neural mechanisms of social touch perception have not been thoroughly investigated in neuropathological populations. Among the most relevant in this context is autism spectrum disorder (ASD), a hereditary neurodevelopmental disorder that is characterized by impairments in social interaction and communication and the presence of restricted, repetitive, and stereotyped patterns of behavior [28]. ASD is often accompanied by an aversion to social touch [29, 30]. Using a limited range of touch stimuli, previous studies have shown that individuals with ASD frequently struggle with both receiving and offering touch [29–31], display reduced empathic resonance to painful touch observation [32], and show diminished neural activity in social brain regions in response to pleasant, gentle touch [33].

While difficulty in interpreting other people’s emotions from non-verbal social cues such as facial [34, 35] and bodily expressions [36, 37] is one of the diagnostic criteria of ASD, the empirical evidence in experimental studies is mixed [38, 39]. At a theoretical level, the socio-communicative impairments of individuals with ASD have often been attributed to impaired ToM abilities [7, 40], as well as to deficits in spontaneous embodied resonance [41–43].

Initial studies showed impaired or delayed development of ToM abilities in ASD, as evidenced by deficits in perspective taking, false belief processing, and emotion recognition [44]. Likewise, at a neural level, individuals with ASD showed attenuated brain activity in the TPJ during various socio-cognitive tasks targeting ToM [45–47]. On the other hand, it has been gradually recognized that many individuals with ASD, especially those with intact intellectual and verbal ability, are able to pass these ToM tasks by means of compensatory sensory strategies and rule-based reasoning [48] despite substantial impairments in spontaneous social communication and interaction in daily life. Moreover, more recent neuroimaging studies revealed that individuals with ASD do show similar brain activation as neurotypical controls during a false belief task and during facial emotional expression recognition [49, 50]. This is where the embodied simulation/resonance account comes into play. According to this account, social impairments in ASD may result from a disability to simulate observed actions and internal states of others via personal sensory and emotional representations [51]. This account is supported by reduced brain activation in the mirror neuron system (MNS) of individuals with ASD during a variety of tasks requiring simulation [41, 42, 52, 53]. Yet, also this “broken mirror theory” of ASD has been criticized based on conflicting evidence showing intact MNS during motor observation in ASD [54]. Thus far, embodied resonance and MNS have mainly been tested in relation to motor mimicry with rudimentary action observation paradigms. Testing this system in relation to a more higher-level socio-affective domain, such as social touch observation, may help clarify whether an individual with ASD spontaneously re-enacts previously acquired sensory experiences to understand other people’s emotional states.

The current study aims at understanding socio-affective touch processing in adults with ASD, both at the behavioral and neural level, using a dynamic stimulus set consisting of videos showing social touch interactions encountered in everyday life. We particularly aim at unraveling whether neural representations of socio-affective touch observation are represented in a cognitive rule-based manner or based on embodied somatosensory resonance. We also investigate to what extent individual differences in socio-affective representations in brain regions relate to the presence of autism symptoms and touch aversion.

## Methods

### Participants

Forty-two men participated in the study, including 21 male adults with a multidisciplinary ASD diagnosis and 21 age-, gender-, and IQ-matched neurotypical (NT) adults (Table 1). Participants with ASD had been diagnosed following DSM-IV or DSM-5 criteria, depending on the year of diagnosis. All were diagnosed by the Expertise Center for Autism at the University Hospitals Leuven. The diagnostic trajectory involves 8 h of patient-contact and assessment, distributed across several sessions, administered by a multidisciplinary team comprising of a psychiatrist, psychologist, social worker, and (optionally) a speech therapist. Assessment encompasses an extensive developmental anamnesis with the patient and his parents, a semi-structured psychiatric interview [56] and/or scoring of the Adult Asperger Assessment inventory [57], an in-depth personality inventory, and an extensive psychological and neuropsychological testing. None of the participants with ASD had comorbid neurological, psychiatric, or genetic conditions, such as epilepsy, traumatic brain injury, or attention-deficit/hyperactivity disorder. On the other hand, healthy adults with no prior diagnosis of ASD were recruited as NT participants through online advertising. None of the NT participants, nor first-degree relatives, had a history of neurological, psychiatric, or medical conditions known to affect brain structure or function. None of the participants in either group took psychotropic medication, and all had normal or corrected-to-normal vision. The ASD participants show above average intelligence and adequate social adaptive functioning (e.g., 11 out of 21 have a regular job and 7 others are students in higher education). There is a partial overlap (5 out of 21 NT participants) between the current NT sample and the data reported in [20]. These five participants were the only ones from the earlier study for whom we had IQ scores and who are male. The sample size was based on previous studies that examined atypical neural representations in clinical populations by means of similar neuroimaging approaches [58, 59]. Moreover, the reliability of behavioral and neural data was thoroughly examined (see below for methods and results), further justifying the adequacy of our sample size.

**Table 1:** Demographics and IQ scores for ASD and NT control groups and descriptive statistics

	ASD		NT		
	M	SD	M	SD	Test statistic
Subject characteristics					
Gender (male/female)	21/0		21/0		
Handedness (right/left)	19/3		18/4		
Age (years)	25.0	4.4	23.9	2.8	*t*(40) = 1, *p = .*32
Total IQ	111.3	14.5	111.5	12.3	*t*(40) = −.31, *p =* .76

*IQ* intelligence quotient assessed with the Wechsler Adult Intelligence Scale (WAIS-IV-NL [55], population average *M* = 100 and SD = 15), *M* mean, *SD* standard deviation. *T* values are from the two-sample *t* test

### Questionnaires

Participants filled out two questionnaires. The *Social Touch Questionnaire* (STQ) assesses individual attitudes towards receiving, offering, and witnessing social touch [60]. The STQ comprises 20 items (e.g., “I generally like it when people express their affection towards me in a physical way”), and participants were asked to respond to each statement on a 5-point scale (1 = strongly disagree, 2 = disagree, 3 = undecided, 4 = agree, 5 = strongly agree). A higher total score indicates a stronger preference for reciprocal touch. Reliability and validity of the STQ are adequate, with a Cronbach’s alpha inter-rater reliability of .89, and moderate to strong correlations (Rs = .42–.74) with 4 out of 5 sub-categories of the Touch Experiences and Attitudes Questionnaire [61].

The *Social Responsiveness Scale for Adults* (SRS-A) is a normed self-report questionnaire measuring a wide range of behaviors characteristic of ASD [62]. The SRS-A comprises 64 items covering subscales for social communication and interaction and for restricted and repetitive patterns of behavior and interests. The SRS-A consists of three subscales measuring social deficits and one measuring restricted and repetitive behavior. A higher total score indicates a higher presence of quantitative autism traits. The reliability and validity of the SRS-A are excellent, with Cronbach’s alpha inter-rater reliability being .80 and strong correlations (Rs = .70) with the Autism Diagnostic Interview-Revised [63].

### Stimuli

**Fig. 1:**
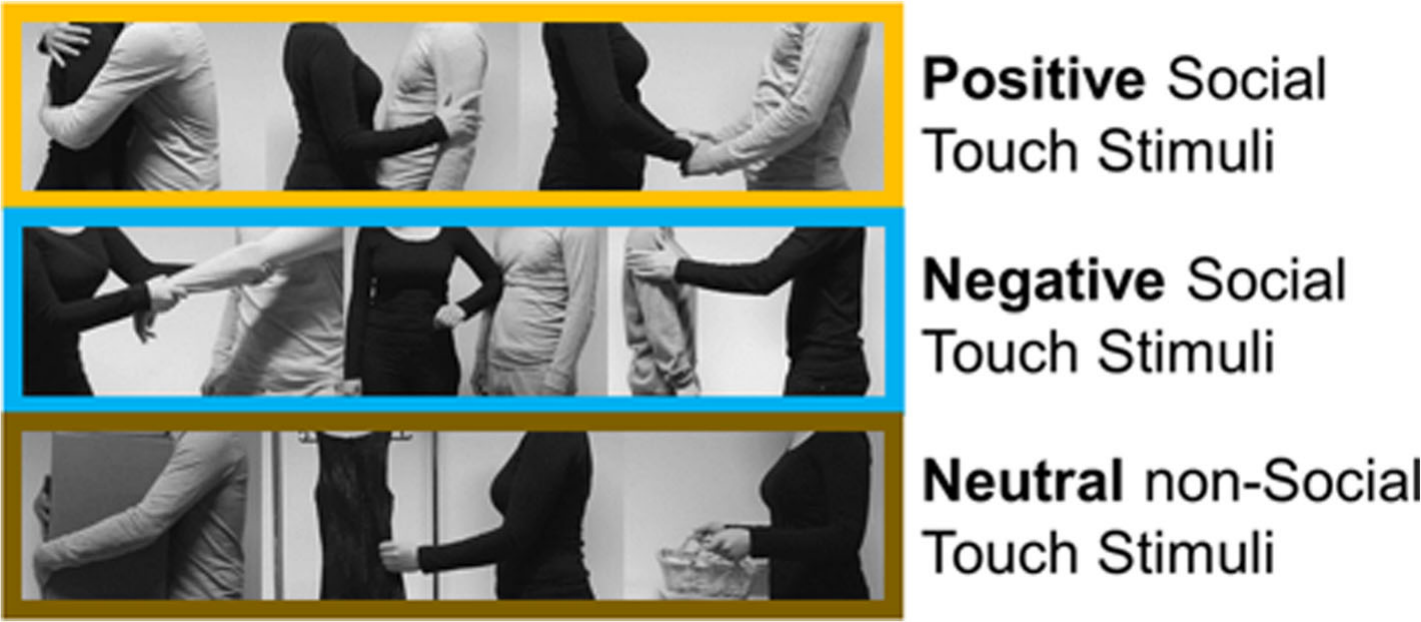
Types of stimuli. The figure shows still frames of exemplary stimuli, showing different types of touch events. Positive, negative, and neutral stimuli are in the first, the second, and the third rows respectively

We used a recently created and well-validated set of 75 greyscale video clips (3 s each) displaying positive (e.g., hugging and holding hands) and negative (e.g., slapping) interpersonal touch interactions as well as neutral object manipulations (e.g., carrying a box). Representative still images of some videos are shown in Fig. 1, and example video clips are available at https://osf.io/8j74m/. The 39 scenes for interpersonal or “social touch” and the 36 scenes for object manipulation or “non-social touch” were closely matched according to the type of physical interactions. For example, the movements involved in hugging another person vs. holding a large box were matched. Various physical parameters from the video sequences were quantified, including pixel-wise intensity, pixel-wise motion energy, and total motion energy [4]. In the current study, the resulting parameters were defined as nuisance covariates in the multiple regression model. A detailed description of the stimuli can be found in our previous study [4]. Psychophysics Toolbox Version 3.0.12 (PTB-3) [64] in MATLAB (R2015a, The MathWorks, Natick, MA) was used for stimulus presentation in all experiments.

### Behavioral rating of valence and arousal

First, participants took part in a behavioral experiment where they viewed all the video clips and reported their subjective feelings of pleasantness (“How pleasant is the touch?” 1—extremely unpleasant, 5—neutral, 9—extremely pleasant) and arousal (“How arousing is the touch?” 1—extremely calm to 9—extremely exciting) in relation to the 75 touch scenes. Each of the 75 stimuli was presented once per session, with a short break in between the two sessions. More details about this experiment can be found in experiment 2 of the previous study [4].

### MRI acquisition

All participants underwent an MRI scanning session consisting of two functional MRI experiments (1 localizer run and 7 main runs) and an anatomical scan. MRI images were acquired on a 3T Philips scanner with a 32-channel coil at the University Hospitals Leuven. Functional imaging was performed with a gapless, echo planar imaging sequence (repetition time (TR) = 2000 ms, echo time (TE) = 30 ms, flip angle (FA) = 90°, field of view (FOV) = 216 × 216 mm, in-plane matrix = 80 × 80, voxel size = 2.7 × 2.7 × 3 mm, 37 slices), with the acquisition of 239 volumes for each run of the main experiment (1673 volumes in total) and 298 volumes for the localizer run.

Structural MR images were collected using a T1-weighted sagittal high-resolution magnetization-prepared rapid gradient echo (MPRAGE) sequence [TR = 9.6 ms, TE = 4.6 ms, FA = 8°, FOV = 250 × 250 mm, in-plane matrix = 256 × 256, voxel size = 0.98 × 0.98 × 1.2 mm, 182 axial slices].

### Main fMRI experiment: observing touch

In the scanner, participants watched the same videos shown during the behavioral experiment while performing an orthogonal attention task (i.e., detecting the color of the shirt of the agent who initiates the touch). The main experiment consisted of 7 runs of 7.8 min each. Note that while structural MRI or resting-state fMRI measures typically involve less than 10 min of scanning time per participant, our multi-voxel pattern analysis (MVPA) study adopts a neuroimaging paradigm that takes about 1 h of continuous scanning time (7 runs) per participant. In each run, the 75 videos were displayed in an optimally designed pseudo-random order in an event-related design. Accordingly, the same touch scenes (e.g., the three slapping scenes) were never displayed consecutively. Every run consisted of 3 blocks, each of which contained a baseline condition displaying a fixation cross (6 s) and 25 trials consisting of video presentation (3 s) and the inter-stimulus interval (ISI, 3 s). All the videos were projected on a screen behind the scanner, and participants viewed them through a mirror mounted on the head coil.

### Localizer fMRI experiment: receiving touch

This experiment was used to localize the (affective) touch-related cortical areas as ROIs within the somatosensory cortex. Note that the current study aimed at investigating the neural representation of observed touch and that the actual touch stimulation only served to confine a touch-related cortical area involving both positive and negative touch. Participants received pleasant (i.e., brush-strokes with a velocity of 5 cm/s) and unpleasant (i.e., rubber band snapping) touch stimulations on the ventral surface of the right and left forearms while lying in the scanner. Pertaining to the pleasant touch, it has been shown that stimulation velocities between 1 and 10 cm/s specifically target unmyelinated C-Tactile afferents, thereby eliciting pleasant touch sensations [65, 66]. The total duration of the localizer run was approximately 10 min. The experiment comprised four randomized blocks (pleasant touch-left arm, pleasant-right, unpleasant-left, and unpleasant-right), and each block consisted of a rest trial (10 s) and eight touch trials (five repetitions of stimulation for 10 s, followed by 6 s of ISI in each trial). A trained experimenter stood next to the scanner and delivered stimulation by following the audio instruction (“start” to start the stimulation, “stop” to end the stimulation). More information can be found in our previous study in NT adults [20].

### Statistical analysis

Statistical inferences were made with one of the following tests depending on the results of the Shapiro-Wilk normality test (with *α* < 0.05): a parametric (e.g., two-tailed one-sample *t* test, two-sample *t* test, and Pearson correlation) vs. non-parametric test (e.g., Wilcoxon signed-rank test, Mann-Whitney *U* test, and Spearman correlation). For the group-based multiple regression analyses, we used a non-parametric permutation test (details are described below). To quantify the group differences, we report Cohen’s *d* effect sizes whenever the null hypothesis is rejected after a *t* test [67]. According to Cohen’s recommendations, an effect size ranging from 0.2 to 0.3 is considered small, values around 0.5 are medium, and values of 0.8 or above are considered large effects. We performed all statistical analyses within the MATLAB (2015a) software environment.

### Behavioral data

The ratings of valence and arousal obtained through the two repetitions were averaged for each participant and stimulus. The ratings of the videos with positive, negative, and non-social touch scenes were analyzed separately to assess whether participants perceived positive touch scenes as relatively more pleasantly, negative touch scenes as unpleasantly, and non-social touch scenes as neutrally. We also compared the arousal ratings of social touch scenes with those of non-social touch scenes. For each of the video categories, we compared the ratings of the ASD vs. NT group in terms of perceived valence and arousal. Lastly, we quantified within- and between-subjects reliability to examine how consistent the ratings were within and between participants in each group (see Additional file 1: within- and between-subjects reliability tests).

In order to use the behavioral data as an independent variable to predict the neural data, we generated an overall affect score integrating the valence and arousal ratings. This was done by calculating the two-dimensional Euclidean distance of valence and arousal ratings for each pair of videos with the Pythagorean theorem, which was first done for each individual and then averaged across individuals. This operation resulted in an affective dissimilarity (distance) matrix. Note that there was a high within- and between-subject consistency of the behavioral valence and arousal ratings (see Additional file 1: intra- and inter-subject consistency of valence and arousal ratings and Additional file 1: Figure S1), justifying the use of a group average affective dissimilarity matrix.

### Functional MRI data analysis

#### Preprocessing, first- and second-level analysis

Imaging data was processed using the Statistical Parametric Mapping software (SPM 12). The standard preprocessing, first- and second-level analyses were implemented. Analysis pipelines are described in detail in Additional file 1 (MRI data preprocessing and first- and second-level analysis).

During the preprocessing phase, we also assessed the head movement of each participant and compared the two groups by using an Artifact Detection and Repair toolbox that calculates a composite measure of scan-to-scan movement. Runs whose maximum frame-wise displacement was greater than the voxel size (3 mm) were discarded (ASD = one run each from two participants; NT = one run from one participant). We found no group difference in the maximum (ASD = 1.38 mm, NT = 1.36, *t*(40) = 0.04, *p* = 0.97) and mean frame-wise head motion displacement (ASD = 0.13 mm, NT = 0.13, *t*(40) = 0.04, *p* = 0.96).

#### Regions of interest

We included the same regions of interest (ROIs) as in our previous study [20]: Brodmann area (BA) 3, BA1, BA2, parietal operculum (PO), insula, middle cingulate cortex (MCC), middle temporal gyrus (MTG), superior temporal gyrus (STG), TPJ, precuneus, BA17, BA18, BA19, BA37, V5, and BA4. All these ROIs are known to be involved in the processing of visually presented social touch scenes in NT adults: vicarious touch processing in the somatosensory network (BA3, BA1, BA2, and PO [68]), the pain network (insula and MCC [69]), the social-cognitive network (MTG, STG, TPJ, and precuneus [12]), and the visual network (BA17, BA18, BA19, BA37, and V5 [70]). The motor cortex (BA4) was also included as motor responses, associated with active button presses, were required during the task in the scanner.

**Fig. 2:**
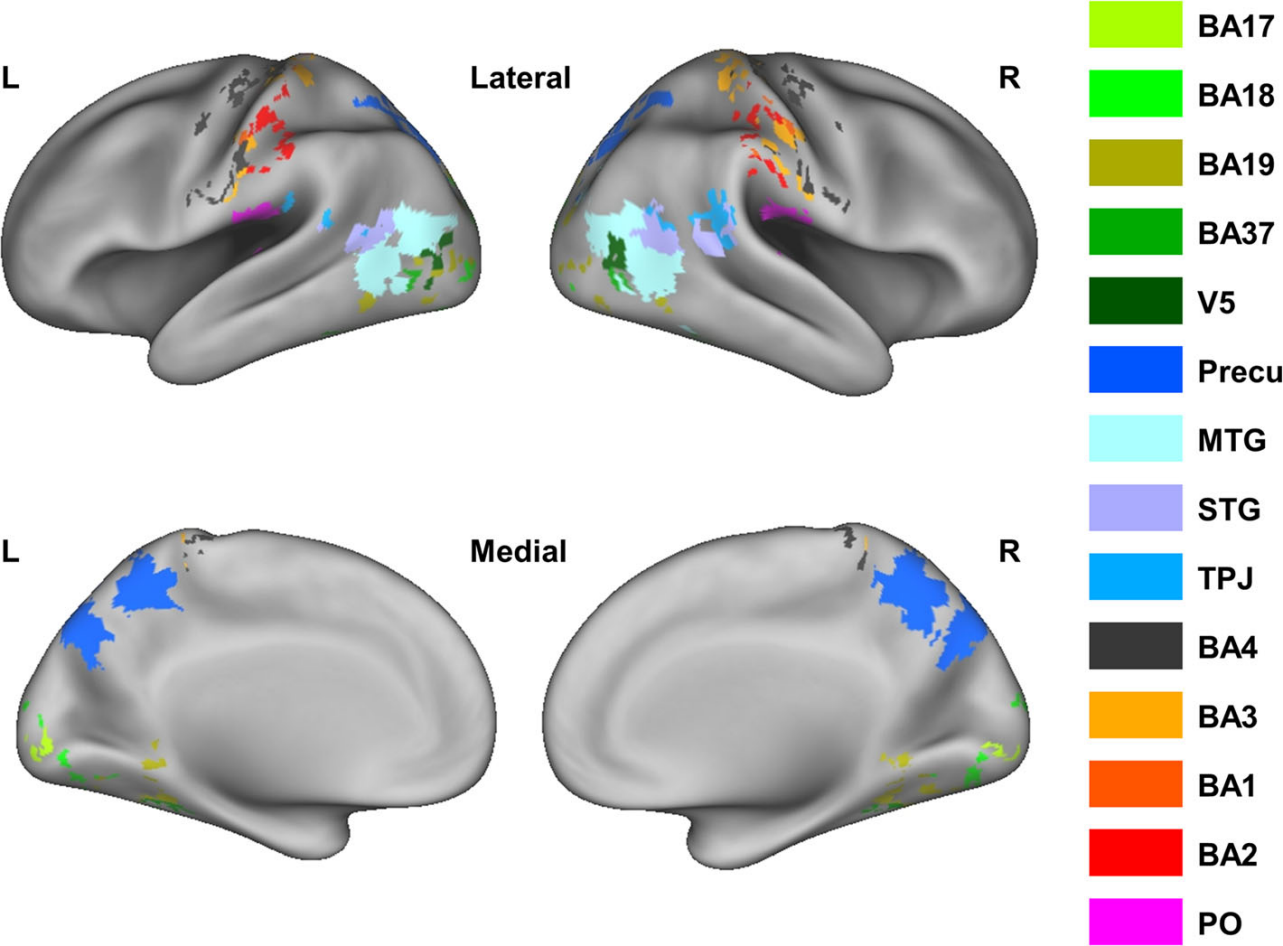
Visualization of ROIs. The figure illustrates the functionally defined ROIs (except anatomically defined BA4) for one example subject, mapped on inflated cortices using the CARET software with PALS atlas. Note that mapping the volume-based data to surface can introduce artefacts

We defined subject-specific ROIs by applying an identical procedure as employed in our previous study [20], including selecting the activated voxels within the anatomical mask for each ROI and trimming the overlapping voxels among the nearby ROIs. When the number of selected voxels was less than 10 per ROI, a more liberal threshold of *p*_uncorrected_ < 0.01 instead of *p*_uncorrected_ < 0.001 was used. Nevertheless, 9 out of 42 participants showed no activation in the insula and 11 out of 42 participants showed no activation in MCC. Accordingly, these two ROIs were not included in the present study. This lack of consistent activation was not surprising, given the low-reliability estimates and limited explanatory power of these same ROIs in our previous study [20]. The low-reliability estimates in these regions found in the previous study do not mean that the current study has low reliability. Instead, it means behavioral data may not be explainable with the neural signals in these regions due to low signal-to-noise ratio in these regions. BA4 was defined based on the anatomical mask only. We did not find any group differences in the size of the ROIs (all *p*_uncorrected_ > 0.06). Mean ROI sizes and the *p* values for individual ROIs are reported in Additional file 1 (mean ROI sizes). With the CARET software [71], the ROIs are shown on the PALS atlas [72] in Fig. 2.

#### Neural representational dissimilarity matrices

For each ROI and each participant, we created neural representational dissimilarity matrices (RDM) capturing the difference in multi-voxel neural response patterns between pairs of videos. For example, if an ROI shows selectivity for the affective valence of the touch scenes, the neural patterns of two differing social touch scenes (e.g., hugging a person vs. slapping a person) will be largely dissimilar. On the other hand, if an ROI does not show this selectivity, the neural patterns will be largely similar across both types of affective interactions.

The “general touch RDM” involved the neural responses for both the social and non-social touch videos and consisted of the pair-wise correlation coefficients of the 75 neural patterns. The “social touch RDM” exclusively involved the neural responses for the social touch videos and consisted of the pair-wise correlation coefficients of the 39 neural patterns. We created these two RDMs per ROI and per participant and tested their reliability by applying an identical procedure as employed in our previous study [20]. The summarized procedure of making RDMs and performing a reliability test can be found in Additional file 1 (neural representational dissimilarity matrices (RDMs) and reliability test for neural data). Note that based on the results of the reliability test, we excluded PO from further analysis as the between-subject variability was too high to conduct group analyses. In total, 42 (21 participants × 2 groups) general touch RDMs with 75 × 75 elements and 42 social touch RDMs with 39 × 39 elements were created per ROI. For the group analysis, we calculated the average of 21 individual RDMs to create each group’s general touch RDM and social touch RDM for each ROI. The RDMs of each ROI were used as dependent variables in each regression model in subsequent analyses.

#### Multiple regression analysis

To investigate which ROIs in which individuals host specific information on the displayed touch scenes, we carried out a series of multiple regression analyses to determine the independent contributions (as represented by the beta coefficients) of each variable of interest to the prediction of the neural data. Prior to this, we vectorized each matrix, took only the upper-diagonal elements, and normalized the vector with a *Z*-score transformation.

In the regression model predicting each group’s general touch RDM, the regressor variables consisted of a binary model of social vs. non-social touch, the motor response made during the task, various physical parameters from the video sequences (pixel-wise intensity, pixel-wise motion energy, and total motion energy), and the type of touch action.

In the regression model predicting each group’s social touch RDM, we replaced the binary model of social vs. non-social touch by each group’s average affective evaluation of social touch (i.e., the affective dissimilarity matrix, see above). We used each group’s average affective dissimilarity matrix in each group’s regression model.

Statistical inferences for each group’s result were based upon a permutation test (1000 iterations), using the same procedure as described in [20]. We randomly shuffled the indices of the vector of neural data and computed the beta coefficients of each independent variable in a multiple regression model applied to the permuted data. We counted the number of times a beta coefficient obtained through this operation was greater than or equal to the observed value in the nonpermuted data. The result of dividing this number by 1000 became the empirical *p* value after being corrected for multiple comparisons with the false discovery rate (FDR).

In order to directly compare the two groups taking into account inter-subject variability, we performed multiple regression on the neural matrices of individual participants in each group and applied either a two-sample *t* test or a Mann-Whitney *U* test to compare the two groups. Against the background of the ToM and embodied simulation accounts (see the “Background” section), we specifically questioned the group difference in the quality of these representations in the core ToM area (TPJ) and the somatosensory cortex (BA3, BA1, and BA2), respectively.

## Results

### Affective responses to social and non-social touch videos

**Fig. 3:**
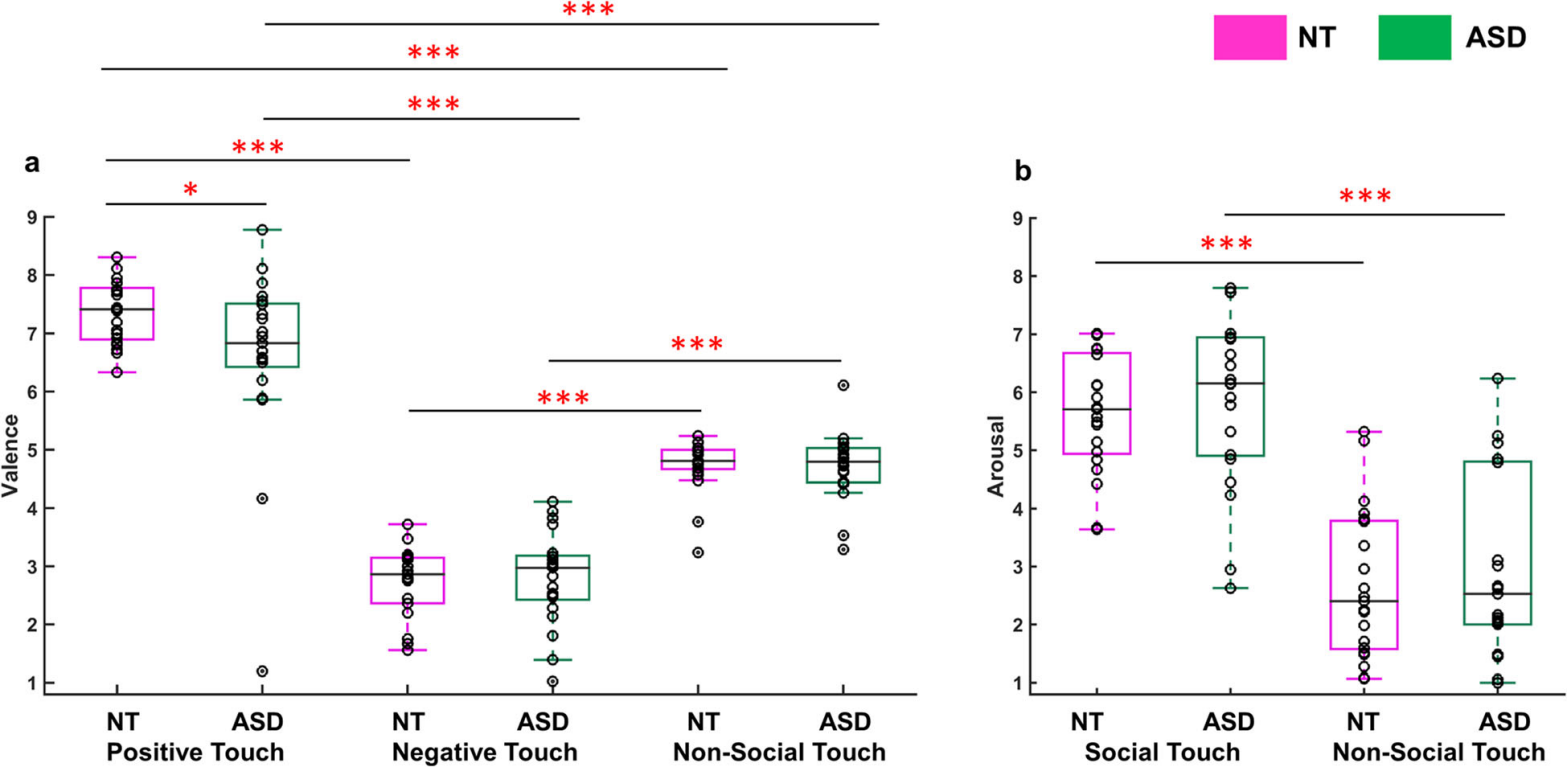
Affective responses to social (positive and negative) and non-social touch stimuli. The boxplots show each group’s valence (**a**) and arousal ratings (**b**) across conditions. The black lines inside each box indicate group medians, and the bottom and top border edges indicate the 25th and 75th percentiles. Data points from individual participants are marked as black circles. The red asterisks indicate statistical significance at *p < 0.05 and ***p < 0.001

Overall, both groups perceived the affective meaning of the touch videos as expected. More specifically, positive touch videos were rated as pleasant (NT, median = 7.4 (the median absolute deviation (MAD) = 0.4); ASD, median = 6.8 (0.6)), negative touch as unpleasant (NT, median = 2.9 (0.3); ASD, median = 3 (0.5)), and non-social touch as neutral (NT, median = 4.8 (0.2); ASD, median = 4.8 (0.2)). Concerning arousal ratings, both groups perceived social touch as exciting (NT, median = 5.7 (0.9); ASD, median = 6.2 (0.8)) and non-social touch as calm (NT, median = 2.4 (0.9); ASD, median = 2.5 (0.6)). Figure 3 shows data points of all individual participants for valence (a) and arousal (b) ratings. In addition, within- and between-subjects reliability tests revealed that participants were consistent in their ratings between the two sessions and were consistent with each other within each group. Additional summary statistics and statistical inference can be found in Additional file 1 (Affective responses to social and non-social touch videos, Intra- and inter-subject consistency of valence and arousal ratings, and Additional file 1: Figure S1).

Regarding the difference between groups, a Mann-Whitney *U* test revealed no group difference in valence ratings of negative (*z* = − 0.21, *p* = 0.83) and non-social touch videos (*z* = 0.42, *p* = 0.68). On the contrary, we observed a significant difference with medium effect size in the rated valence of the positive videos between the two groups (*z* = 1.99, *p* = 0.046, effect size *d* = 0.65), indicating that participants with ASD perceived positive social touch, such as a hug, as relatively less pleasant. Although outlying data points were observed in the valence ratings (Fig. 3a), the Mann-Whitney *U* test can robustly handle this as it is based upon medians rather than means. Neither group differed in their arousal ratings of social (z = − 0.97, *p* = 0.33) and non-social touch videos (z = − 0.40, *p* = 0.69).

### Social touch preference and its association with quantitative autism traits

Individuals with ASD showed a less positive appreciation towards giving, receiving, and witnessing social touch in daily life (STQ: *M*_ASD_ = 56.8, SD_ASD_ = 13.2), as compared to the NT group (*M* = 69.2, SD = 9.6; *t*(40) = 3.21, *p* = 0.003, *d* = 1.07). As expected, individuals with ASD show a higher number of autism traits, as compared to NT individuals (SRS-A: *M*_ASD_ = 63.8, SD_ASD_ = 11.6; *M*_NT_ = 51.9, SD_NT_ = 8.9; *t*(40) = − 3.72, *p* < 0.001, *d* = 1.15). This group difference was significant on each of the social deficit subscales: social awareness (*M*_ASD_ = 60.9, SD_ASD_ = 11.6; *M*_NT_ = 50.2, SD_NT_ = 10.1; *t*(40) = − 3.18, *p* = 0.003, *d* = 0.98), social communication (*M*_ASD_ = 61.4, SD_ASD_ = 11.2; *M*_NT_ = 51.4, SD_NT_ = 8.2; *t*(40) = − 3.33, *p* = 0.002, *d* = 1.03), and social motivation (ASD *M*_ASD_= 60.5, ASD_SD_ASD_ = 11.6; NT *M*_NT_ = 51.4, SD_NT_ = 8.8; *t*(40) = − 2.88, *p* = 0.006, *d* = 0.89). Overall, we found large to very large group differences in social touch preference and quantitative autism traits.

**Fig. 4:**
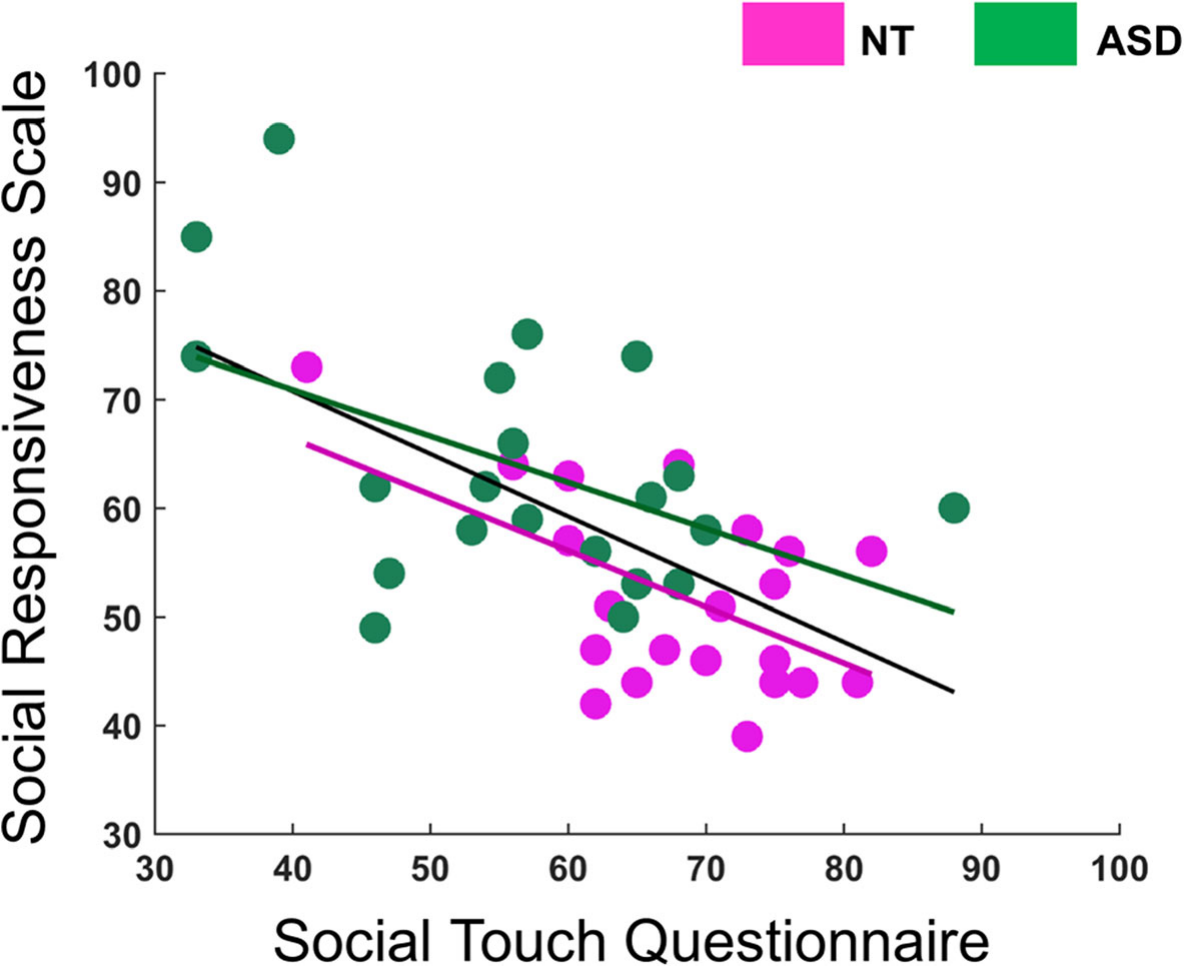
Social touch preference and its association with quantitative autism traits. The green, pink, and black trend lines indicate the association in the ASD group (*r* = − 0.62, *p* < 0.001), the NT group (*r* = − 0.55, *p* = 0.009), and across all participants (*r* = − 0.48, *p* = 0.03), respectively

The correlational analysis revealed a negative linear association between individual differences in social touch preference and the number of autism traits experienced by an individual (all participants *r* = − 0.62, *p* < 0.001; NT group *r* = − 0.55, *p* = 0.009; ASD group *r* = − 0.48, *p* = 0.03, Fig. 4). Similar associations were present for each of the social deficit subscales (Additional file 1: Table S1). Together, our results confirm that individuals with ASD present social impairments and exhibit a higher degree of social touch avoidance. Furthermore, the participants avoiding social touch seem to exhibit stronger social impairment characterized by atypical social awareness, communication, and motivation, implying a tight link between social touch aversion and autism symptom severity.

### Univariate neural responses to observed and felt touch

Two-sample *t* tests revealed no significant group difference in neural responses for the contrast of social vs. non-social touch videos and vice versa (*p*_FWE_ < 0.05). Mean group effects of social vs. non-social touch contrasts are shown in Additional file 1 (Neural responses to observed and felt touch, Additional file 1: Figure S2 and Table S2 for detailed information such as MNI coordinates of peak activity). Similarly, no significant group difference in neural responses for felt touch was found (not at *p*_FWE_ < 0.05 and not at *p*_uncorrected_ < 0.02) (Additional file 1: Neural responses to observed and felt touch, Additional file 1: Figure S3 and Table S3).

### Neural representations underlying observed social vs. non-social touch processing

**Fig. 5:**
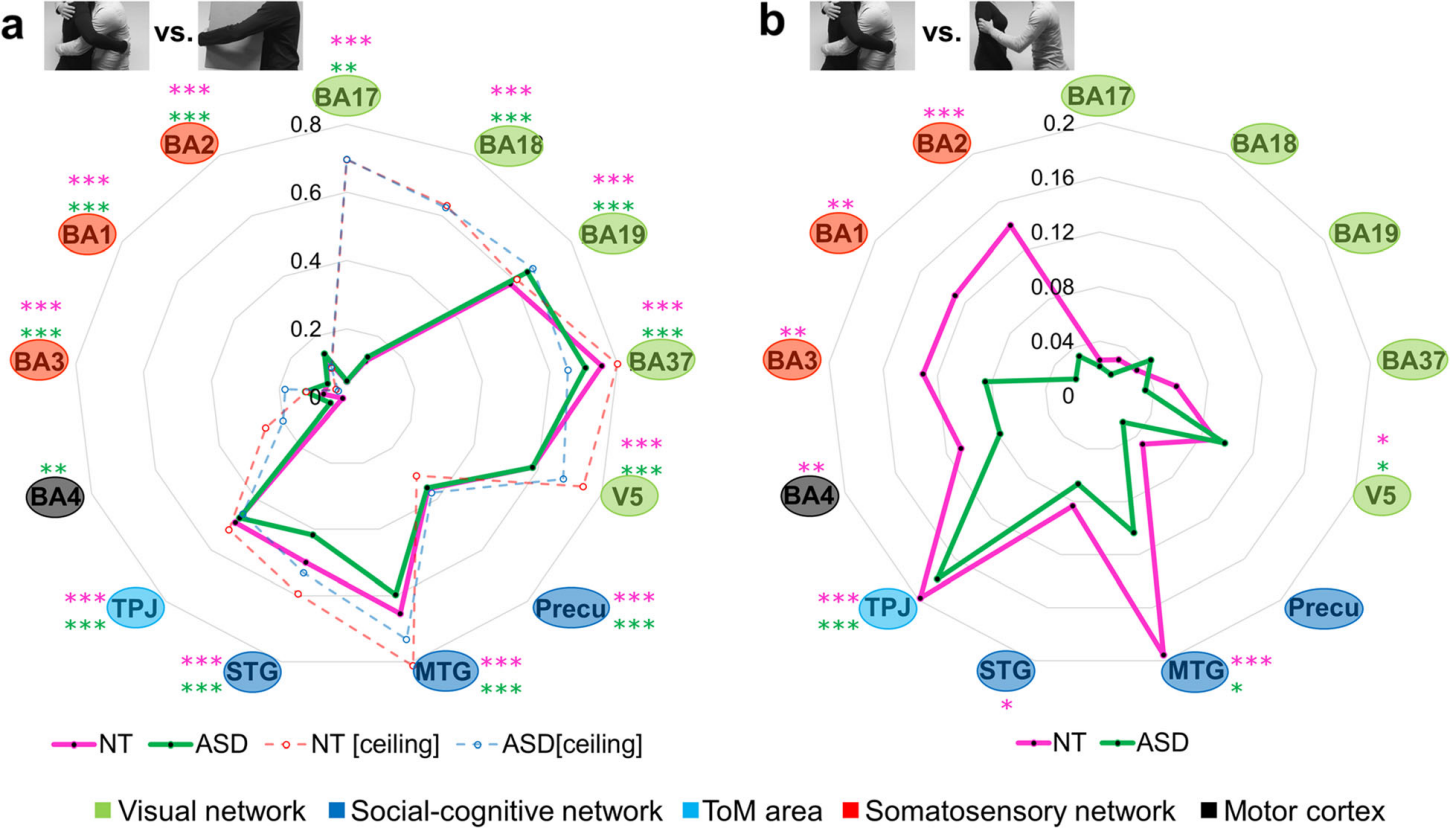
Neural representations of the social versus nonsocial distinction and of affective meanings in touch scenes. Radar charts were used to plot the results (a pink line for the NT and a green line for the ASD group). Each of the 13 ROIs, ordered according to the implied brain network as indicated by the color of the surrounding circles, forms an individual axis. The node (anchor) on the spoke (axis) represents the beta coefficient of each ROI. **a** The beta coefficient from the multiple regression model in which the neural patterns of each of the ROIs were predicted based on the social vs. non-social factor. **b** The beta coefficient from the multiple regression model predicting the neural patterns based on perceived overall affect. The asterisks indicate the statistical significance (beta higher than zero) determined by the permutation tests at *p < 0.05, **p < 0.01, and ***p < 0.001 in the NT (pink) and ASD group (green). In **a**, we additionally plotted the correlation coefficient representing the noise ceiling of the neural data, derived from the reliability test (a red dashed line for the NT group and a blue dashed line for the ASD group)

In line with earlier research by others [73] and ourselves [20], we expected that on top of this univariate selectivity for social vs. non-social touch, observed in both groups, there would also be high multi-voxel selectivity for the distinction between social and nonsocial touch videos. The multiple regression analysis confirmed that almost every implicated ROI represents the distinction between social and non-social touch scenes (11 of 13 *p* values < 0.001 for both groups), even after controlling for the effects of all the other regressor variables (e.g., low-level visual features and motor response). Figure 5a displays the main results, and more details can be found in Additional file 1 (Neural representations underlying observed social vs. non-social touch processing). Importantly, no significant group differences in neural selectivity for this distinction were found in the core ToM area (TPJ (*t*(40) = − 0.67, *p* = 0.50)) and the somatosensory areas (BA3 *z* = 0.93, *p* = 0.35; BA1 *z* = − 0.05, *p* = 0.96; BA2 *z* = − 0.31, *p* = 0.76), indicating well-preserved neural selectivity in the ASD group for the social vs. non-social aspects shown in touch actions of others.

Note that these and all the following multi-voxel analyses require a reproducible signal, and between-group comparisons are easier to interpret if the reliability is comparable between the two groups. To assess whether there may be group differences in the reliability of the neural data in the TPJ and the somatosensory cortex, we calculated values of the leave-one subject-out correlations within each group (correlating the neural data of one subject with the group averaged neural data after excluding this subject). Our results demonstrated that there was no group difference in the reliability of neural patterns in the four ROIs that are central to the tested hypotheses (BA3 *t*(40) = − 1.75, *p* = 0.09; BA1 *t*(40) = 0.40, *p* = 0.69; BA2 *t*(40) = − 0.24, *p* = 0.81; TPJ *t*(40) = 1.40, *p* = 0.17).

In sum, our results suggest that the brains of individuals with and without ASD can equally distinguish whether another person’s touch actions comprise social interactions or not, and this rudimental social processing is implemented across multiple brain areas including visual, somatosensory, and social regions.

### Neural representations underlying dimensions of overall affect in social touch observation

By combining the valence and arousal dimensions, we obtained a measure of overall affect conveyed in the social touch scenes. We investigated how this affective meaning of social touch is implemented in the brain when participants watch the interpersonal touch actions of others. In the NT group, statistically significant representations of overall affect were observed in V5 (*β* = 0.09, *p* = 0.04), MTG (*β* = 0.20, *p* < 0.001), STG (*β* = 0.08, *p* = 0.04), TPJ (*β* = 0.20, *p* < 0.001), the motor cortex (*β* = 0.11, *p* = 0.01), and the somatosensory cortex (BA3 *β* = 0.13, *p* = 0.002; BA1 *β* = 0.13, *p* = 0.004; BA2 *β* = 0.14, *p* < 0.001) (see the pink line in Fig. 5b and Additional file 1: Table S4). In the ASD group, however, significant representations of overall affect were found in V5 (*β* = 0.10, *p* = 0.05), MTG (*β* = 0.10, *p* = 0.03), and TPJ (*β* = 0.18, *p* < 0.001), but not in the somatosensory cortex (BA3 *β* = 0.08, *p* = 0.08; BA1 *β* = 0.02, *p* = 0.32; BA2 *β* = 0.03, *p* = 0.32), STG (*β* = 0.07, *p* = 0.12), and the motor cortex (*β* = 0.08, *p* = 0.09) (the green line in Fig. 5b and Additional file 1: Table S4).

Comparing both groups in terms of the strength of neural selectivity for the fine-grained socio-affective information in the core ToM area and the somatosensory cortex, we found no significant difference between the two groups in TPJ (*t*(40) = 1.04, *p* = 0.30), but significantly weaker representations in BA1 (*t*(40) = 3.06, *p* = 0.004, *d* = 0.94) and BA2 (*t*(40) = 2.45, *p* = 0.02, *d* = 0.76) in the ASD group. Effect sizes indicate a large to very large group difference in the quality of affective representations in BA1 and BA2. No significant group difference was found in BA3 (*t*(40) = 0.90, *p* = 0.37). Similar results were observed when age or mean frame-wise head motion displacement was included as a covariate in an analysis of covariance model. The present results indicate that both groups are able to represent subtle socio-affective nuances of observed social touch interactions. However, whereas individuals with ASD only represent this information in high-level visual areas (V5, MTG) and cognitively oriented ToM areas (i.e., TPJ), NT individuals additionally represent this information in a more embodied somatosensory format (BA3, BA1, and BA2). The neural representations of other factors, such as motor response and low-level visual features, were additionally examined. No group difference was found either in visual or in motor processing (Additional file 1: Figure S4).

### Neural correlates of individual differences in touch avoidance and autistic traits

In our previous study in NT adults, we demonstrated that individual differences in the strength of neural representations of socio-affective touch in the somatosensory cortex were associated with individual differences in the attitude towards social touch in daily life [20]. Here, we extend these findings and connect the neuroscientific findings with core pervasive autistic traits. Note that the results described below were observed only when the two groups were merged and analyzed together.

### Social touch preference

When correlating the scores on the Social Touch Questionnaire (STQ) with the beta coefficients indexing the quality of the overall affect representations in somatosensory cortex, the results indicate that a more positive attitude towards social touch is significantly associated with higher quality of overall affect representations in BA1 (rS = 0.43, *p* = 0.008) and BA2 (rS = 0.32, *p* = 0.04). Individual differences in affect representations in BA3 (rS = 0.08, *p* = 0.64) were not linked to the individual attitude towards social touch. Similar results were observed when age and mean frame-wise head motion displacement were assigned as a covariate in a rank partial correlation model. Our results suggest that the functional organization and vicarious emotional sensitivity of the somatosensory cortex (i.e., BA1 and BA2) of individuals with a positive attitude towards receiving, witnessing, and providing social touch may differ from the one of individuals who show social touch aversion.

### Social impairments

Likewise, the correlations between SRS-A scores and the strength of overall affect representations in somatosensory cortex revealed that individual differences in social responsiveness were significantly associated with the distinctness and specificity of overall affect representations in BA1 (rS = − 0.38, *p* = 0.02) and BA2 (rS = − 0.45, *p* = 0.003). Individual differences in affect representations in BA3 (rS = − 0.12, *p* = 0.46) were not linked to individual differences in self-reported autistic traits. Again, similar results were observed when age and mean frame-wise head motion displacement were assigned as a covariate variable in a rank partial correlation model.

Together, our results imply that the presence and the quality of affective representations of visually observed touch interactions in mid-to-high level somatosensory cortex (i.e., BA1 and BA2) show an association with quantitative autism features and personal attitudes towards social touch. In particular, individuals who show higher social impairments tend to show a higher degree of social touch aversion, and both these characteristics are associated with less robust socio-affective representations in the mirror-somatosensory system during the observation of social touch.

## Discussion

Our study investigates the neural basis of socio-affective touch observation in adults with ASD compared to well-matched NT adults. In particular, we sought to clarify to what extent social impairments and touch aversion in ASD may be linked to aberrant cognitive representations of the affective aspects of touch (due to impaired ToM abilities) or to an inability to re-enact pre-acquired somatosensory experiences (due to deficits in embodied somatosensory resonance). Using fMRI-based MVPA methods and a well-defined set of stimuli, we were able to pinpoint the atypicality of ASD in processing complex touch scenes containing multidimensional information (visual, somatosensory, and socio-affective). Our study provides novel evidence that adults with ASD specifically lack a differentiated somatosensory resonance when observing complex social touch interaction of others, despite a high degree of commonalities with NT adults in other aspects of neural information processing.

Adults with ASD rated the socio-affective touch scenes fairly similarly as NT adults, and both groups showed a high intra- and inter-subject consistency in their ratings. The only difference was in the perception of the positive touch scenes, such as a hug, which the adults with ASD rated less pleasantly as compared to NT adults. While the effects are subtle, the results of this computer-based behavioral experiment are consistent with those of the questionnaire, which also revealed a significantly lower preference for receiving and observing social touch in daily life in adults with ASD. Similar to Voos et al. [33], we also found an association between individual differences in social touch preference and the number of self-reported autism traits, while using different measurement instruments.

At the neural level, we found surprisingly high similarities between the two groups. Using both univariate and multivariate analysis approaches, intact neural selectivity for the social vs. non-social distinction of touch scenes was observed, in multiple brain regions including the ToM area and the somatosensory cortex. Although previous studies have shown that individuals with ASD may process social stimuli in an atypical manner [74, 75], the neural patterns associated with social vs. non-social touch scenes may still be distinctive as long as both conditions are perceived sufficiently differently from each other. This was indeed the case, also in the ASD sample, as illustrated by the ratings of valence and arousal in Fig. 3. Accordingly, the current findings indicate that social impairments in ASD are not simply due to an inability to distinguish between social and non-social information.

Likewise, similar (univariate) neural activation in response to actual touch stimulation was observed in both groups in the current study. This observation contrasts with previous findings showing diminished neural response to affective touch events, especially to gentle brushstrokes, in individuals with ASD or in individuals scoring high on autistic traits [33, 76, 77]. Possibly, this discrepancy may be due to the administration of both positive and negative touch stimulation in our study, unlike the aforementioned studies which only delivered positive touch.

Strikingly, neural commonality with the NT group was even evident with regard to more delicate and fine-grained socio-affective processing. Individuals with ASD did represent subtle and differentiated information on the overall affect of socio-affective touch interactions in the TPJ, the most classical “social cognition ToM module”, suggesting intact social cognitive reasoning. Functional abnormalities, such as reduced neural activation, in this region have been attributed to the impairments in social cognitive reasoning in various disorders, such as schizophrenia, [78] bipolar disorder [79], and ASD [45–47]. In the current study, however, we did not find any evidence that individuals with ASD exhibit functional abnormalities in the TPJ. On the contrary, the presence of intact fine-grained affective touch representations in this region suggests that individuals with ASD are capable of mentalizing the affective meaning of observed social touch interactions. For decades, impaired ToM ability has been put forward as the primary cause of socio-communicative impairments in ASD [7, 40, 44]. However, several recent studies have shown that intellectually and verbally gifted individuals with ASD, like the ones in our sample, successfully pass ToM tasks, possibly by using compensatory strategies [48–50].

Despite the typical ToM involvement in our ASD sample and despite the numerous behavioral and neural commonalities among both groups, we did observe significant and very specific differences in the more automatic and spontaneous processing of socio-affective touch interactions. Unlike NT adults, the ASD group did not show affective touch representations in mid-to-high level somatosensory areas (i.e., BA1 and BA2), indicating a lack of embodied resonance in relation to others’ bodily experiences. Our findings thereby extend studies demonstrating reduced empathic resonance to a painful touch experience of others as reflected in weaker mu suppression in ASD [32]. A lack of embodiment of others’ emotional state—not only painful sensations but also joyful ones—as revealed in the current study, further supports the argument that social difficulties in ASD may involve a lack of embodied simulation [80].

Lastly, building upon our previous study in NT adults [20], the current study provides evidence that individuals with stronger social touch avoidance or with more autistic traits experience diminished embodied somatosensory resonance with others. These findings extend recent studies that demonstrated an association between the level of activation in the somatosensory system during the observation of touch and inter-individual differences in empathy [24–27].

### Limitations

The current study instigates new questions that will require further research. In particular, while our ASD sample showed a clear dissociation between intact socio-affective representations in TPJ vs. severely affected and absent representations in mid-to-high level somatosensory areas (with large group differences), it should be noted that the current study only included a selective subsample of male adults with ASD showing average to above-average intelligence and no language deficits. Although the homogeneity of this sample allowed controlling for confounding factors such as age, IQ, and gender, future studies may benefit from the inclusion of children, women, and more severely affected individuals, including individuals with low IQ, who may not mobilize compensatory cognitive strategies. Indeed, it remains an open question whether also these individuals would show intact rule-based ToM representations. Likewise, as the present study used a relatively effortless task, it remains to be seen whether intact ToM processing would still be in place when a task requires more higher-level cognitive exertion (e.g., understanding the meaning of touch based on the social norm and culture) [11].

## Conclusions

The current study provides strong support for the impaired embodied simulation account of ASD [41–43]. Accordingly, the less positive attitude towards reciprocal touch in ASD may be a consequence of the deficient automatic emotional resonance and the resulting increase in cognitive processing load during such interactions. Gallese and Sinigaglia [81] nicely illustrated the different formats of representations, and its impact, with the analogy of a route description: “Just as a map and a series of sentences might represent the same route with a different format, so might mental representations have partly overlapping contents while differing from one another in their format (e.g., bodily instead of propositional)” (p. 517). Crucially, while the same information can be represented in different formats, its utility is constrained by the format. Evidently, representing in a bodily format an emotion, such as disgust or pain, or a sensation, such as being touched, is different from representing them in a propositional format [81]. According to our findings and to the extent that TPJ may be a more cognitive and rule-based ToM module, individuals with ASD may not have access to the bodily format of the affective touch representations, but they have access to the propositional format. As a result, the depth of understanding and experiencing the state of others (and themselves) may differ between the two groups (i.e., “knowing” vs. “knowing and feeling”), which is also related to alexithymia in ASD [82]. The current findings may also motivate to reconsider how cognitive behavioral therapies designed to enhance mentalizing capacities of individuals with ASD can be complemented by more physical and bodily intervention strategies (e.g., mirror imitation therapy [83, 84];) that directly target the deficient emotional resonance in clinical practice.

## Supplementary information

**Additional file 1: Figure S1.** Intra-subject, and inter-subject consistency. **Table S1.** The strength and direction of a linear relationship between the social touch behavior and total and three subscale scores of SRS-A. **Figure S2.** Brain areas involved in social vs. non-social touch observation. **Table S2.** Brain areas activated during the observation of social touch compared to non-social touch and vice versa. **Figure S3.** Brain areas showing increased neural activation for receiving touch. **Table S3.** Brain areas activated when receiving touch compared to resting. **Table S4.** The beta coefficients of the social/non-social and overall affective dimensions for all ROIs and both groups. **Figure S4.** Neural representations of motor responses made during the task (A) and of the pixel-wise intensity of the video frames (B).

## Data Availability

Data that support the findings of this study are available through the Open Science Framework (https://osf.io/6ktwc/) for scientific use.

## Abbreviations

ASD: Autism spectrum disorder
BA: Brodmann area
GLM: General linear model
IQ: Intelligence quotient
MAD: Median absolute deviation
MCC: Middle cingulate cortex
MNS: Mirror neuron system
MTG: Middle temporal gyrus
MVPA: Multi-voxel pattern analysis
NT: Neurotypical
PO: Parietal operculum
RDM: Representational dissimilarity matrix
ROI: Region of interest
RSA: Representational similarity analysis
SD: Standard deviation
SRS-A: Social Responsiveness Scale for Adults
STG: Superior temporal gyrus
STQ: Social Touch Questionnaire
ToM: Theory of mind
TPJ: Temporoparietal junction
