# Supplementary material for: Intact neural representations of affective meaning of touch but lack of embodied resonance in autism: a multi-voxel pattern analysis study
Source: Mol Autism. 2019 Nov 27;10:39. doi: 10.1186/s13229-019-0294-0 (PMC6881998; doi:10.1186/s13229-019-0294-0)
Supplement: Supplementary file 1 — Additional file 1: Figure S1. Intra-subject, and inter-subject consistency. Table S1. The strength and direction of a linear relationship between the social touch behavior and total and three subscale scores of SRS-A. Figure S2. Brain areas involved in social vs. non-social touch observation. Table S2. Brain areas activated during the observation of social touch compared to non-social touch and vice versa. Figure S3. Brain areas showing increased neural activation for receiving touch. Table S3. Brain areas activated when receiving touch compared to resting. Table S4. The beta coefficients of the social/non-social and overall affective dimensions for all ROIs and both groups. Figure S4. Neural representations of motor responses made during the task (A) and of the pixel-wise intensity of the video frames (B). [file 13229_2019_294_MOESM1_ESM.docx]

**Additional file**

**Additional Methods**

**Within- and between-subjects reliability tests**

We conducted within- and between-subjects reliability tests on resulting ratings for each group. For the within-subject reliability tests, we calculated the Spearman correlation for the ratings of 75 stimuli between the two test sessions for each participant. For the between-subjects reliability test, a split-half correlation method was used, measuring the correlation between the ratings of one half of the participants and those of the other half of the participants.

**MRI data preprocessing**

Preprocessing steps include 1) slice timing correction, 2) realignment of functional images to the mean image of the first run (the images from the localizer experiments were also aligned to this mean image), 3) registration of the anatomical image to the functional images, 4) segmentation which provides forward deformation fields, 5) normalization process which warps all structural and functional images to a Montreal Neurological Institute (MNI) space with a re-sampling size of 3 x 3 × 3 mm based on forward deformation fields, and 6) spatial smoothing. Gaussian kernels with a 5 mm full-width at half maxima (FWHM)) was chosen as the smoothing parameter, except for the second-level univariate group analysis (8 mm FWHM).

**First- and second-level analysis**

The first-level (subject-level) analysis was carried out with a standard general linear model (GLM). Delta functions were used to model the regressors for the main runs (observing touch) while boxcar functions were used for the localizer run (receiving touch). The regressors of interest differed across four GLMs.

1) The first GLM was created for a second-level univariate group analysis and was fitted to the 8 mm FWHM smoothed preprocessed imaging data. This GLM contained three predictors, i.e. social, non-social, and baseline condition, referring to the social touch videos, the non-social touch videos, and a fixation cross, respectively. The resulting data were used for standard random-effect group-level whole brain analyses were conducted. We identified significantly activated voxels in the “social minus non-social touch observation” contrast, and vice versa, and we compared brain activation between the two groups for each contrast. All statistical maps were thresholded at P _FWE_ < 0.05.

2) The second GLM was created for a representational similarity analysis (RSA) and was fitted to the 5 mm FWHM smoothed preprocessed imaging data. This GLM contained 75 predictors, i.e., one regressor for each video stimulus. We used the resulting 75 estimated beta-values as input for MVPA to construct a subject-specific neural dissimilarity matrix.

Two more GLMs were additionally fitted for defining ROIs. 3) The third GLM (fitted to the 5 mm FWHM smoothed data from the main experiment) again contained the three predictors social touch observation, non-social touch observation, and baseline (fixation cross). We used the contrast of all the touch videos versus baseline to identify the majority of ROIs, except for the touch-related ROIs. 4) The last GLM (fitted to the 5 mm FWHM smoothed data from the localizer experiment) contained three predictors: pleasant touch, unpleasant touch, and rest condition. We used the contrast of both touch conditions versus rest to identify the first-hand touch-selective cortical regions as ROIs.

**Mean ROI sizes**

Mean ROI sizes after trimming and p-values for group comparisons were as follows: BA3 = 81 (ASD = 78; TD = 83; p = 0.67); BA1 = 21 (ASD = 19; TD = 23; p = 0.17); BA2 = 67 (ASD = 62; TD = 71; p = 0.38); PO = 109 (ASD = 97; TD = 120; p = 0.21); Precuneus = 447 (ASD = 414; TD = 480; p = 0.44); MTG = 404 (ASD = 355; TD = 453; p = 0.06); STG = 197 (ASD = 176; TD = 219; p = 0.26); TPJ = 116 (ASD = 104; TD = 127; p = 0.43); BA17 = 88 (ASD = 86; TD = 90; p = 0.57); BA18 = 290 (ASD = 283; TD = 296; p = 0.66); BA19 = 247 (ASD = 233; TD = 260; p = 0.29); BA37 = 94 (ASD = 82; TD = 107; p = 0.06); V5 = 42 (ASD = 41; TD = 43; p = 0.50); BA4 = 386 voxels. Here, reported p-values were not adjusted for multiple comparisons corrections. The p-values corrected for the false discovery rate (FDR) for multiple comparisons are all above 0.42.

**Neural representational dissimilarity matrices (RDMs)**

The procedure of constructing RDMs include the following steps: 1) extracting beta-values ​​of all voxels in each ROI for each stimulus, 2) per run normalizing the beta-values for each voxel in each ROI by subtracting the average beta-value over all conditions, 3) generating multi-voxel patterns for each stimulus by averaging the normalized beta-values ​​of seven runs, 4) generating two symmetric matrices (i.e., 75 x 75 general touch matrix and 39 x 39 social touch matrix) per ROI by computing the Pearson correlation coefficients between multi-voxel patterns of each possible pair combination of stimuli (RSA, [1]), 5) lastly, transforming the matrix into RDM by subtracting correlation coefficients from 1.

**Reliability test for neural data**

Two reliability tests were performed – one for each participant group separately – to test whether an RDM of each ROI contains a reliable signal.

First, by comparing the diagonal of RDMs (diagonal = comparison of a condition with itself) with the non-diagonal, we verified whether multi-voxel patterns of a particular ROI are worth further analysis [2]. The procedure included: 1) randomly splitting the seven runs into two halves, 2) calculating the average matrix of each, 3) correlating the two, 4) placing the resulting correlation coefficients of the within-conditions and the between-condition correlations on the diagonal and non-diagonal elements respectively of a 75 × 75 square matrix, 5) repeating the aforementioned steps for 100 times, and 6) calculating the average of 100 square matrices per ROI and group. To measure whether the multi-voxel patterns contain a meaningful signal, we compared the correlation coefficients of the diagonal cells of the matrix created with the aforementioned methods with those of the shuffled matrix of which indices were randomly rearranged. Statistical inferences were based upon a permutation test (1000 times of iterations). We counted the number of times a shuffled matrix contained an averaged diagonal value that was greater than or equal to that of an original matrix ​​and then divided the number by 1000. The result of dividing this number by 1000 became the empirical p-value after being corrected using the FDR for multiple comparisons (cf. the number of ROIs). Based on this analysis, for both groups, we did not exclude any ROI from further analysis.

Second, with the split-half between-subject correlational method [3], we estimated the maximum regression coefficient we could expect in predicting the neural data with other variables while taking into account between-subject variability in the multi-voxel patterns. 21 RDMs of each group were randomly split into two halves and averaged. We vectorized each matrix, took only the upper-diagonal elements, and correlated a resulting vector of one matrix with that of another matrix. Finally, we adjusted the resulting correlations with the Spearman-Brown formula (2 × r/(1 + r)). We repeated the aforementioned steps 100 times, each time randomly designating participants into two sub-groups. In the end, the results are averaged per ROI and group. P-values were corrected using the FDR for multiple comparisons (cf. the number of ROIs). Based on the results, we excluded PO (no correlation between participants, r = 0.009) from the further analysis.

**Additional Results**

**Affective responses to social and non-social touch videos**

A Wilcoxon signed-rank test revealed that rated valence was significantly higher for the positive touch videos than the negative ones in both groups (NT, z = 4.02, p < 0.001; ASD, z = 3.98, p < 0.001). In line with this, in both groups, we observed significant differences in the rated valence between positive videos and non-social ones (NT, z = 4.01, p < 0.001; ASD, z = 3.29, p < 0.001) and negative ones and non-social ones (NT, z = - 4.02, p < 0.001; ASD, z = - 3.98, p < 0.001). The rated arousal was significantly higher for the social touch videos than the non-social ones in both groups (NT, z = 4.01, p < 0.001; ASD, z = 4.01, p < 0.001).

**Intra- and inter-subject consistency of valence and arousal ratings**

The results revealed that participants were consistent in their ratings between the two sessions (NT, valence median rS = 0.90, p < 0.001; arousal median rS = 0.85, p < 0.001; ASD, valence median rS = 0.85, p < 0.001; arousal median rS = 0.77, p < 0.001). Fig. S1A illustrates data points of individual participants’ correlations between the two sessions for valence ratings. A Mann-Whitney U test revealed that two groups show no difference in within-subject consistency for valence (z = 0.98, p = 0.33) and arousal ratings (z = 1.43, p = 0.15), indicating that individuals with ASD rated the valence and the arousal of the stimuli as coherently as NT adults across two sessions.

Concerning inter-subject consistency, the results revealed high consistency for valence (NT, median rS = 0.85, p < 0.001; ASD, median rS = 0.74, p < 0.001) and arousal ratings (NT, median rS = 0.73, p < 0.001; ASD, median rS = 0.69, p < 0.001) within the group (Fig. S1B). Unlike intra-subject consistency, however, significant differences in inter-subject consistency for the valence (z = 9.58, p < 0.001) and arousal ratings (z = 2.32, p = 0.02) were observed, suggesting more heterogeneity of ratings in the ASD group despite the high consistency.


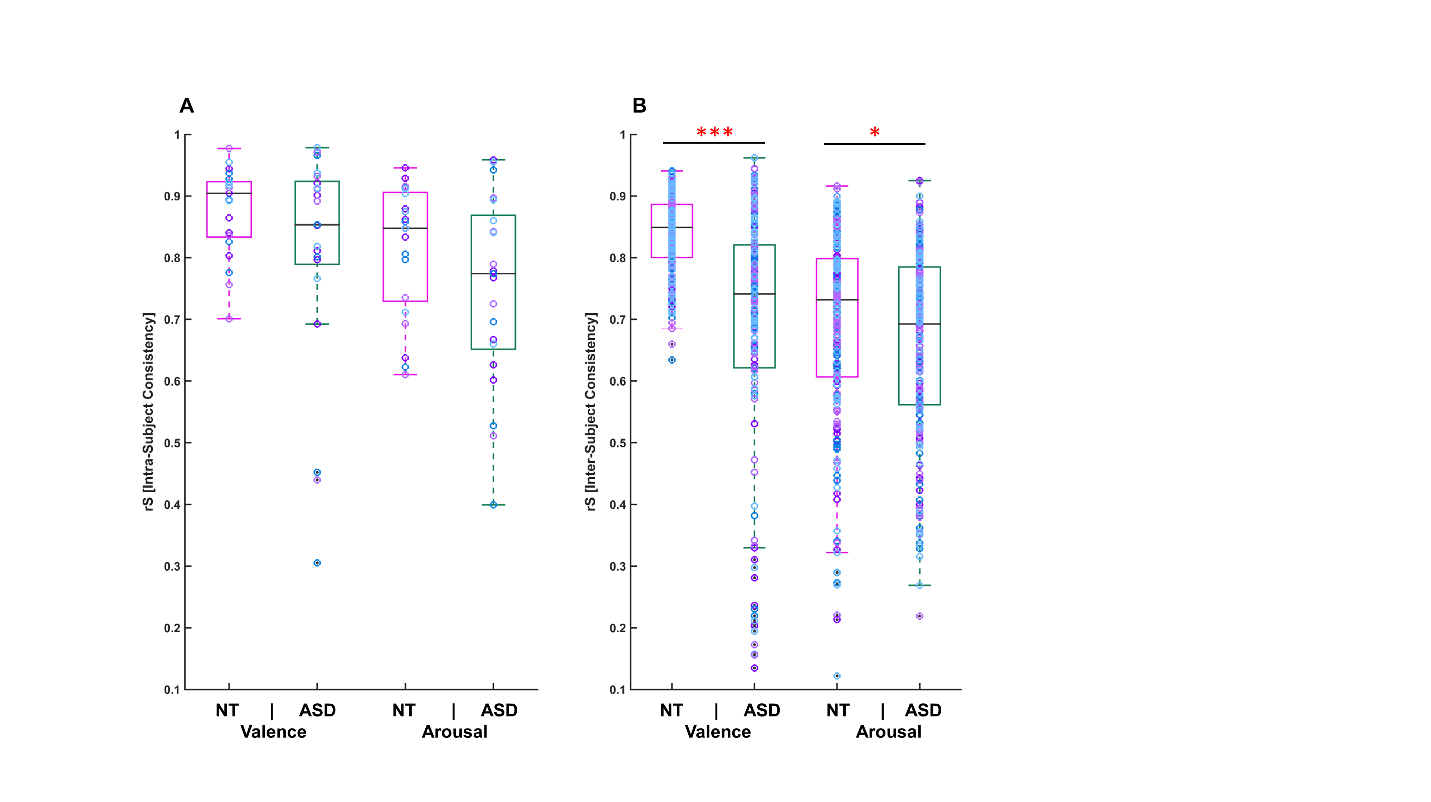


**Fig. S1. Intra-subject, and inter-subject consistency.** The figure illustrates intra- (A) and inter-subject consistency (B) on valence and arousal ratings in both groups. The central black lines inside of the boxes indicate the group medians of Spearman correlations. The bottom and top border edges of the boxes illustrate the 25^th^ and 75^th^ percentiles respectively; the whiskers illustrate the range of the rank correlation coefficients within 99.3 % coverage. All data points are additionally marked as purple/sky-blue circles. The black dots dawn outside of the whiskers indicate the outliers. The single red asterisk indicates the statistical significance at p < 0.05 and three red asterisks show statistical significance at p < 0.001.

**Table S1** The strength and direction of a linear relationship between the social touch behavior and total and three subscale scores of SRS-A. Bold text indicates the significant association between the two variables.

|  | ASD | | NT | | Both | |
| --- | --- | --- | --- | --- | --- | --- |
|  | *r* | *P* | *r* | *P* | *r* | *P* |
| Total SRS-A scores  Social Awareness  Social Communication  Social Motivation | **-.48**  -.4  **-.59**  -.36 | .03  .08  .005  .11 | **-.55**  -.42  **-.54**  **-.67** | .009  .06  .01  .001 | **-.62**  **-.47**  **-.63**  **-.49** | .001  .002  .001  .001 |

**Neural responses to observed and felt touch**

**Observing touch**

A second-level univariate analysis across all participants revealed that the current results of the contrast between social and non-social touch observation conform well to our previous findings [4]. Increased brain activity was observed in multiple brain areas, including limbic (i.e., the anterior insular cortex) and social brain regions (e.g., the superior temporal gyrus (STG), the TPJ, the medial prefrontal cortex (MPFC), and the precuneus) during the observation of social touch as compared to non-social touch (thresholded at p _FWE_  <   0.05). On the other hand, the fusiform gyrus (FuG), a high-level visual region implicated in object recognition, was relatively more active during the observation of non-social touch videos (involving a person handling an object).


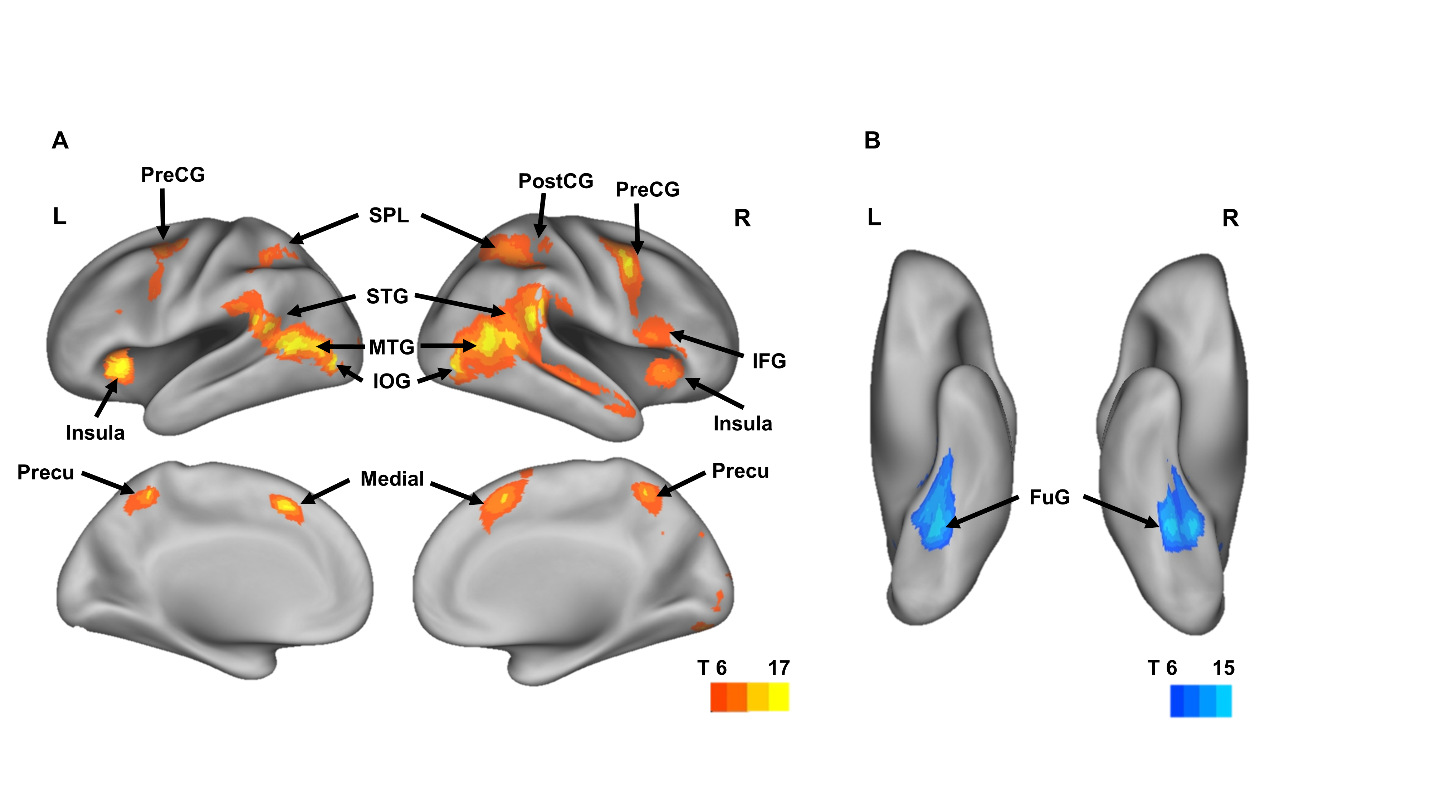


Fig. S2. Brain areas involved in social vs. non-social touch observation. Figure (A) shows the significant differences (univariate random-effects between-subject analysis) in the contrast of social touch minus non-social touch while figure (B) shows the reverse contrast of non-social touch minus social touch (*P*_FWE_ < 0.05, k= 60). The two groups did not differ in univariate activity. L = left hemisphere, R = right hemisphere, PreCG = precentral gyrus, PostCG = postcentral gyrus, SPL = superior parietal lobe, STG = superior temporal gyrus, MTG = middle temporal gyrus, IOG = inferior occipital gyrus, Medial = medial prefrontal cortex, Precu = precuneus, FuG = fusiform gyrus, T = t-values

**Table S2 Brain areas activated during the observation of social touch compared to non-social touch and vice versa.**

| **Social Touch Observation > Non-Social Touch Observation** | | | | | | |
| --- | --- | --- | --- | --- | --- | --- |
|  | Peak X | Peak Y | Peak Z | T(40) | P | N Voxels |
| R MTG/IOG/AnG | 48 | -58 | 11 | 20.5 | 0.000 | 3642 |
| R STG/SMG/AnG | 60 | -40 | 20 | 17.3 | 0.000 | / |
| R IOG | 48 | -73 | -1 | 15.7 | 0.000 | / |
| R MFG/PreG | 42 | 5 | 44 | 15.7 | 0.000 | 1194 |
| R AIns/FO/IFG | 33 | 26 | 2 | 12.1 | 0.000 | / |
| R MFG/PreG/SFG | 33 | -1 | 53 | 10.3 | 0.000 | / |
| L AIns/FO/IFG | -30 | 23 | 5 | 13.4 | 0.000 | 627 |
| L PreG/MFG | -39 | -4 | 53 | 10.5 | 0.000 | / |
| L PreG/MFG | -45 | 2 | 35 | 8.1 | 0.000 | / |
| R SMC/SFG | 9 | 14 | 53 | 13.2 | 0.000 | 539 |
| L SMC/MCgG | -6 | 14 | 47 | 12.4 | 0.000 | / |
| R SMC/SFG | 9 | 5 | 62 | 8.05 | 0.000 | / |
| R LiG | 18 | -82 | -10 | 12.2 | 0.000 | 211 |
| R LiG | 12 | -88 | -4 | 9.7 | 0.000 | / |
| R OCP | 15 | -94 | 8 | 8.7 | 0.000 | / |
| L OCP | -21 | -94 | 11 | 8.7 | 0.000 | 62 |
| **Non-Social Touch Observation > Social Touch Observation** | | | | | | |
|  | Peak X | Peak Y | Peak Z | T(40) | P | N Voxels |
| L FuG/LiG | -27 | -49 | -10 | 16.6 | 0.000 | 476 |
| L FuG/Hippo/ParaHioppo | -30 | -34 | -16 | 11.68 | 0.000 | / |
| R LiG/FuG | 24 | -46 | -10 | 15.8 | 0.000 | 433 |
| R FuG/ Hippo/ParaHioppo | 33 | -19 | -25 | 6.7 | 0.001 | / |

**Receiving touch**

As with the observed touch paradigm, the second-level univariate analysis revealed that the current results of felt touch minus rest condition conform well to our previous findings [4]. Increased brain activity was observed in multiple brain areas including limbic (i.e., the posterior insular cortex and the middle cingulate gyrus) and somatosensory areas (i.e., the postcentral gyrus and the parietal operculum) when receiving touch as compared to rest (p _FWE_ <   0.001) (Fig. S3. and Table S3). No group differences were found in this experiment, which in the current context was mainly used as a localizer to help define the somatosensory regions.


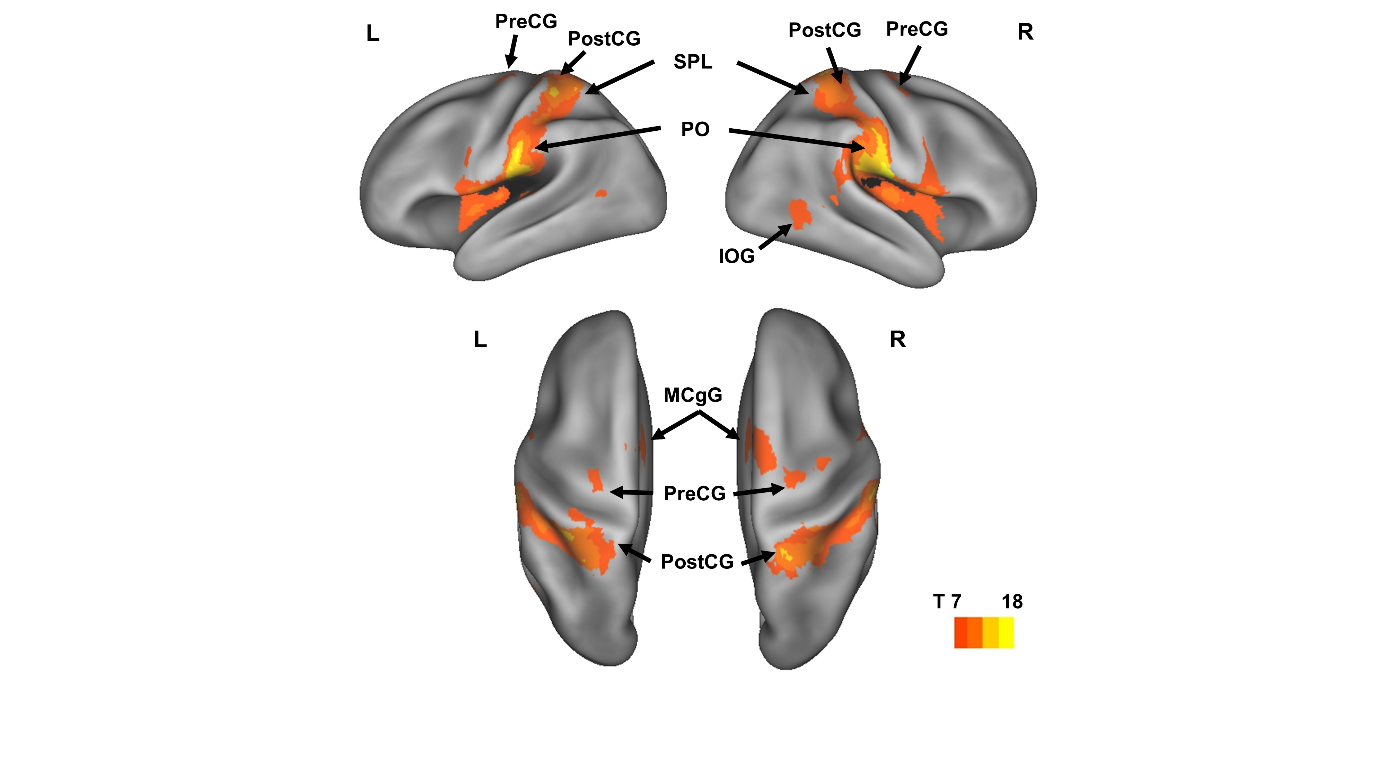


**Fig. S3.** **Brain areas showing increased neural activation for receiving touch.** The figure shows the mean group effects of felt touch minus rest (p FWE < 0.001, k= 60). We mapped the contrast result on inflated cortices with PALS atlas using CARET software. MCgG = middle cingulate gyrus, PO = parietal operculum.

**Table S3 Brain areas activated when receiving touch compared to resting.**

| **Receiving Touch > Rest** | | | | | | |
| --- | --- | --- | --- | --- | --- | --- |
|  | Peak X | Peak Y | Peak Z | T(40) | P | N Voxels |
| PO | 57 | -19 | 20 | 18.2 | 0.000 | 2778 |
| SPL/PostG | 24 | -43 | 68 | 14.2 | 0.000 | / |
| SPL/PostG | 51 | -25 | 35 | 13.2 | 0.000 | / |
| PO | -51 | -25 | 17 | 16.9 | 0.000 | 2293 |
| PostG/SMG | -57 | -22 | 29 | 16.4 | 0.000 | / |
| PostG/SPL | -36 | -37 | 56 | 13.6 | 0.000 | / |
| Cerebellum | 18 | -70 | -22 | 12.8 | 0.000 | 359 |
| Cerebellum/FuG | -27 | -55 | -25 | 12.5 | 0.000 | / |
| Cerebellum/FuG | 27 | -58 | -25 | 12.4 | 0.000 | / |
| SFG/SMG | 9 | -1 | 71 | 10.4 | 0.000 | 806 |
| SMG/MCgG | 0 | -1 | 53 | 9.6 | 0.000 | / |
| SFG/SMG | -9 | -4 | 74 | 9.4 | 0.000 | / |
| MTG/IOG/MOG | -54 | -70 | 8 | 9.5 | 0.000 | 66 |

**Neural representations underlying observed social versus non-social touch processing**

Both NT and ASD participants showed high multi-voxel selectivity for the distinction between social and nonsocial touch scenes. The neural patterns of BA37 (NT β = 0.75, p < 0.001; ASD β = 0.71, p < 0.001) and of MTG (NT β = 0.65, p < 0.001; ASD β = 0.60, p < 0.001) were the best predicted by the social vs. non-social variable. Furthermore, in both groups, the social vs. non-social variable could explain changes in the distributed patterns of neural activity in the somatosensory regions (e.g., BA 2 NT β = 0.13, p < 0.001; ASD β = 0.14, p < 0.001), implying intact representations of the rough social vs. non-social subdivision of the observed touch scene in somatosensory regions of the ASD group. Likewise, in both groups, TPJ showed high neural selectivity for the social vs. non-social contrast. Table S4 contain a complete list of results.

Additionally, the results of between-subject reliability test revealed that the amount of variation in the neural data explained by the social vs. non-social model reached almost the noise ceiling in the areas BA37, MTG, TPJ, BA1, BA2 and BA3 (e.g., BA 37 of the NT group r (the ceiling) = 0.80 and β = 0.75). In contrast, the social vs. non-social model (β = 0.05) did not account for much of the neural patterns in the early visual cortex, although these areas exhibit high noise ceilings (e.g., BA17 of the NT group r = 0.70). It implies that other (more low-level) regressor variables may have a greater impact on explaining the neural data of these areas (Fig. S4B).

**Table S4 The beta coefficients of the social/non-social and overall affective dimensions for all ROIs and both groups.**

|  | **Social/Non-Social** | | | | | | **Affect** | | |
| --- | --- | --- | --- | --- | --- | --- | --- | --- | --- |
|  | NT | ASD | | | | | NT | ASD | |
| BA17 | 0.05** | 0.05** | | | | | 0.03 | | 0.02 |
| BA18 | 0.12*** | | | | | 0.13*** | 0.03 | | 0.02 |
| BA19 | 0.58*** | | | | 0.64*** | | 0.03 | | 0.05 |
| BA37 | 0.75*** | | | 0.71*** | | | 0.06 | | 0.03 |
| V5 | 0.58*** | | 0.58*** | | | | 0.09* | | 0.10* |
| Precuneus | 0.36*** | 0.36*** | | | | | 0.05 | | 0.03 |
| MTG | 0.65*** | 0.60*** | | | | | 0.20*** | | 0.10* |
| STG | 0.50*** | 0.42*** | | | | | 0.08* | | 0.07 |
| TPJ | 0.49*** | 0.47*** | | | | | 0.20*** | | 0.18*** |
| BA4 | 0.01 | 0.05** | | | | | 0.11** | | 0.08 |
| BA3 | 0.07*** | 0.12*** | | | | | 0.13** | | 0.08 |
| BA1 | 0.07*** | 0.07*** | | | | | 0.13** | | 0.02 |
| BA2 | 0.13*** | 0.14*** | | | | | 0.14*** | | 0.03 |

Asterisks denote FDR-corrected p values from the permutation test (* p < 0.05, ** p < 0.01, *** p < 0.001).


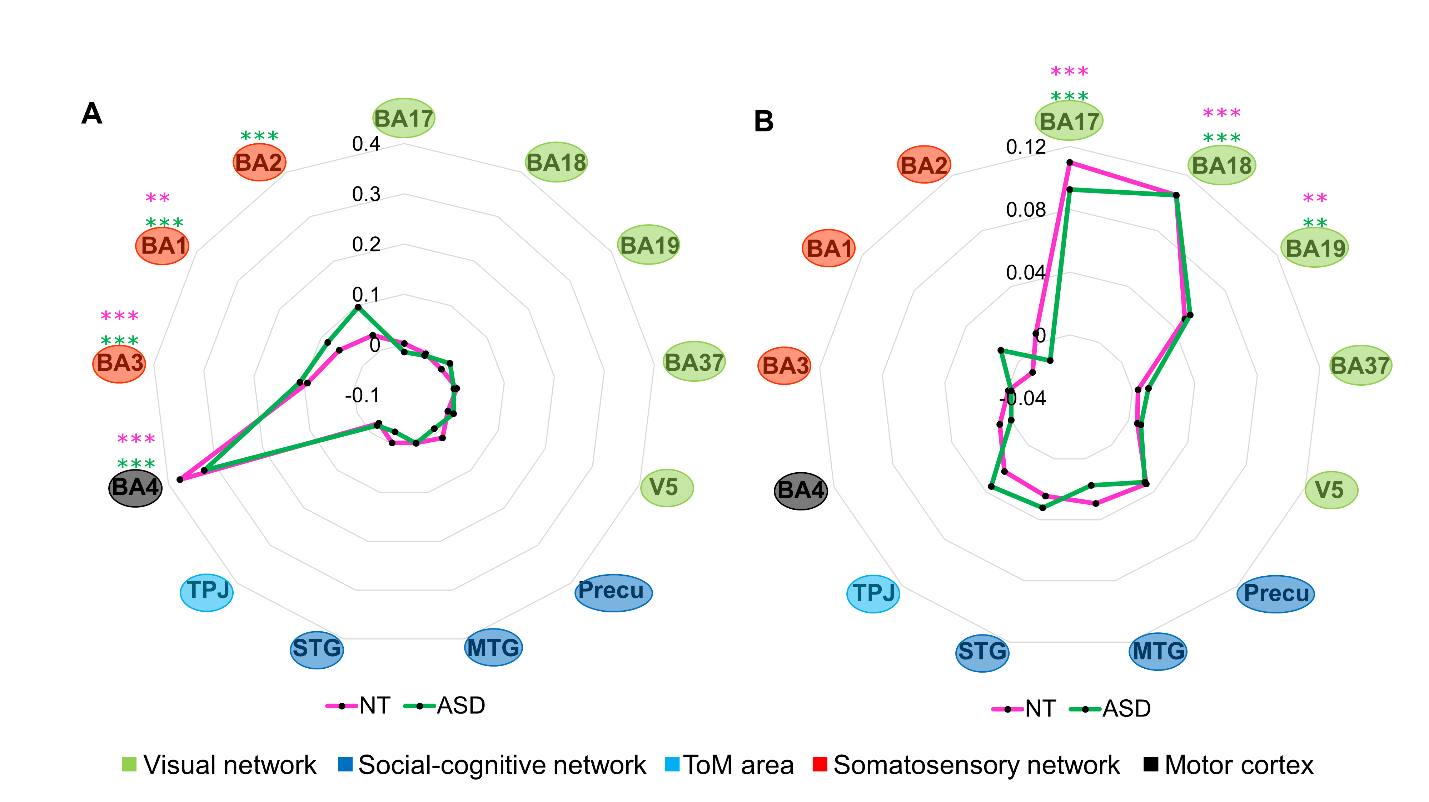


Fig. S4. Neural representations of motor responses made during the task (A) and of the pixel-wise intensity of the video frames (B). Radar charts were used to plot the results of 13 ROIs in both groups (a pink line used for the NT group and a green line used for the ASD group) on the same graph. Each of the 13 ROIs forms an individual axis that is radially arranged. The ROIs are ordered according to the brain network they belong to. The color of the circles surrounding the name of the ROI indicates the implied network (visual network in light green, social-cognitive network in blue, ToM area in sky blue, somatosensory network in red, and motor cortex in black). The node (anchor) on the spoke (axis) represents the beta coefficient of each ROI. The figures show the beta coefficient of each ROI from the multiple regression model in which the neural patterns of the ROIs were predicted based on motor reponses made during the task (A) and based on pixel-wise intensity of the videos (B), respectively. The asterisks indicate the statistical significance determined by the permutation tests at **P* < 0.05, ***P* < 0.01, and ****P* < 0.001 in the NT (pink) and ASD group (green). The figure indicates the motor and visual representations in the motor cortex and the occipital lobe respectively in both groups, highligting the intact motor and visual processing in ASD.

**References**

1. Kriegeskorte N, Mur M, Bandettini PA. Representational similarity analysis – connecting the branches of systems neuroscience. Front Syst Neurosci. 2008; 2:4.

2. Op de Beeck HP, Torfs K, Wagemans J. Perceived shape similarity among unfamiliar objects and the organization of the human object vision pathway. J Neurosci. 2008; 28:10111–23.

3. Bracci S, Op de Beeck H. Dissociations and Associations between Shape and Category Representations in the Two Visual Pathways. J Neurosci. 2016; 36:432–44.

4. Lee Masson H, Van De Plas S, Daniels N, Op de Beeck H. The multidimensional representational space of observed socio-affective touch experiences. Neuroimage. 2018; 175:297–314.
